# A Role of Sp1 Binding Motifs in Basal and Large T-Antigen-Induced Promoter Activities of Human Polyomavirus HPyV9 and Its Variant UF-1

**DOI:** 10.3390/ijms18112414

**Published:** 2017-11-14

**Authors:** Ugo Moens, Xiaobo Song, Marijke Van Ghelue, John A. Lednicky, Bernhard Ehlers

**Affiliations:** 1 Molecular Inflammation Research Group, Department of Medical Biology, Faculty of Health Sciences, University of Tromsø, 9037 Tromsø, Norway; 2 Host Microbe Interaction Research Group, Department of Medical Biology, Faculty of Health Sciences, University of Tromsø, 9037 Tromsø, Norway; xiaobo.song@uit.no; 3 Department of Medical Genetics, University Hospital Northern-Norway, 9038 Tromsø, Norway; marijke.van.ghelue@unn.no; 4 Department of Environmental and Global Health, College of Public Health and Health Professions, University of Florida, Gainesville , FL 32603, USA; jlednicky@phhp.ufl.edu; 5 Division 12, Measles, Mumps, Rubella and Viruses Affecting Immunocompromised Patients, Robert Koch Institute, 13353 Berlin, Germany; EhlersB@rki.de

**Keywords:** large T antigen, luciferase, mutation, non-coding control region, Sp1

## Abstract

Human polyomavirus 9 (HPyV9) was originally detected in the serum of a renal transplant patient. Seroepidemiological studies showed that ~20–50% of the human population have antibodies against this virus. HPyV9 has not yet been associated with any disease and little is known about the route of infection, transmission, host cell tropism, and genomic variability in circulating strains. Recently, the HPyV9 variant UF-1 with an eight base-pair deletion, a thirteen base-pair insertion and with point mutations, creating three putative Sp1 binding sites in the late promoter was isolated from an AIDS patient. Transient transfection studies with a luciferase reporter plasmid driven by HPyV9 or UF1 promoter demonstrated that UF1 early and late promoters were stronger than HPyV9 promoters in most cell lines, and that the UF1 late promoter was more potently activated by HPyV9 large T-antigen (LTAg). Mutation of two Sp1 motifs strongly reduced trans-activation of the late UF1 promoter by HPyV9 LTAg in HeLa cells. In conclusion, the mutations in the UF1 late promoter seem to strengthen its activity and its response to stimulation by HPyV9 LTAg in certain cells. It remains to be investigated whether these promoter changes have an influence on virus replication and affect the possible pathogenic properties of the virus.

## 1. Introduction

Polyomaviruses, a family of small, double-stranded DNA viruses that infects mammals, birds, and fish, while polyomavirus-like sequences have also been found in reptiles and invertebrates [1,2,3]. In 1971, the first two human polyomaviruses BK polyomavirus (BKPyV) and JC polyomavirus (JCPyV) were described and named after the initials of the patients from which they were isolated [4,5]. It was not until 2007 that a third human polyomavirus (Karolinska Institute polyomavirus, KIPyV) was isolated [6], and since then, another 11 not previously reported polyomaviruses have been detected in human samples. These are: Washington University polyomavirus (WUPyV), Merkel cell polyomavirus (MCPyV), HPyV6, HPyV7, *Trichodysplasia spinulosa* polyomavirus (TSPyV), HPyV9, Malawi polyomavirus (MWPyV), Saint Louis polyomavirus (STLPyV), HPyV12, New Jersey polyomavirus (NJPyV), and Lyon IARC polyomavirus (LiPyV) [1,7]. All human polyomaviruses share high sequence similarity in their coding region, whereas the non-coding control region (NCCR) that encompassed the origin of replication and the transcription control sequences for the early and late genes is highly variable [1]. Also, between isolates of a human polyomavirus species, hypervariability in the NCCR is observed. The changes in the NCCR of specific human polyomavirus affect the life cycle of the virus and may be associated with the pathogenic properties of the virus, as was convincingly shown for BKPyV and JCPyV [8,9]. Major mutations and rearrangements in the NCCR have been reported in other human polyomaviruses [10], but the effects on the properties of the virus have been scarcely examined [11,12].

HPyV9 was first detected in the serum from a renal transplant patient under immunosuppressive treatment [13], and shortly after from the facial surface of a Merkel cell carcinoma patient [14]. Seroprevalence of HPyV9 varies between ~6–39% in children (<1 month-11 years), ~18–47% in healthy adults (>16 years of age), and reaches 70% in individuals aged 80 years and older [15,16,17,18,19,20,21,22,23,24,25]. Whether different HPyV9 variants are circulating in the human population has scarcely been investigated. The genomes of both isolates differed by just two nucleotides. Recently, a third HPyV9 variant was isolated from peripheral blood monocytes of an AIDS patient [26]. This variant, designated HPyV9 UF-1 (hereafter referred to as UF-1), differs by two amino acids in the VP2/3 proteins relative to the originally isolated HPyV9 genomes [13,14], and has several changes in its non-coding control region (NCCR). The biological implications of these mutations on the viral life cycle are not known as permissive cell systems for virus propagation are lacking [25,26].

In an effort to determine the biological importance of the differences in NCCR between HPyV9 and UF-1, we examined their relative early and late promoter strength in seven different human cell lines and studied the effect of HPyV9 LTAg on the early and late promoter activities. The possible role of additional Sp1 sites in UF-1 NCCR when compared to the HPyV9 NCCR in promoter activity was also investigated.

## 2. Results

### 2.1. The Basal UF-1 Early and Late Promoter Activity is Higher than that of HPyV9 in Most Cell Lines Tested

**Figure 1:**
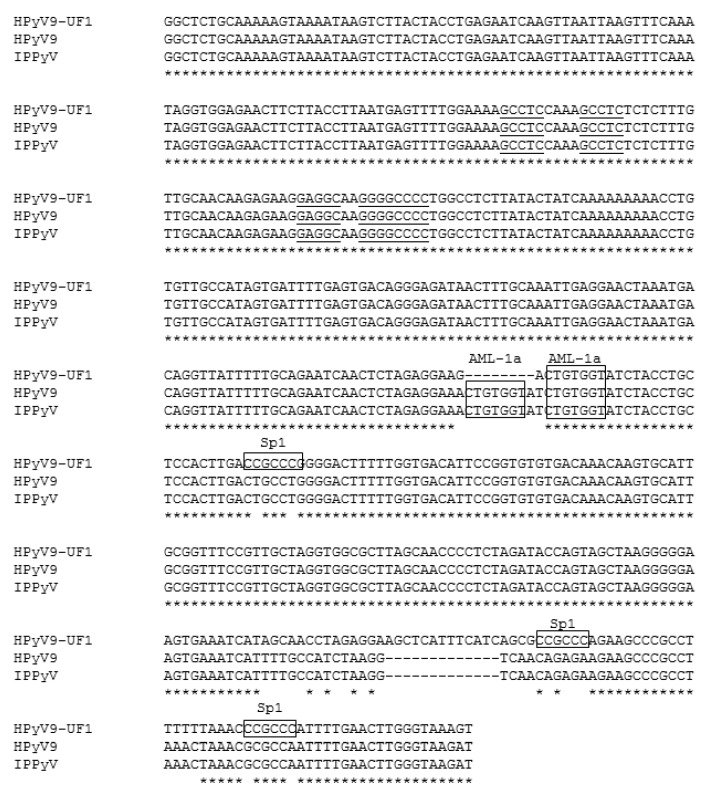
Alignment of the non-coding control region (NCCR) of human polyomavirus 9 (HPyV9), IPPyV and UF-1. IPPyV was the name given to the HPyV9 variant described by Sauvage et al. [14]. Indels are indicated by dashes, asterisk show identical nucleotides. The putative Sp1 and AML-1a motifs are shown in a frame, while the putative HPyV9 LTAg-binding motifs are underlined. The NCCR is depicted in an early-to-late direction.

The NCCR of the originally described HPyV9 isolates [13,14] is identical, while the UF-1 variant contains several point mutations, a 13-base pair insertion and an 8-base pair deletion as compared to HPyV9 (Figure 1).

**Figure 2:**
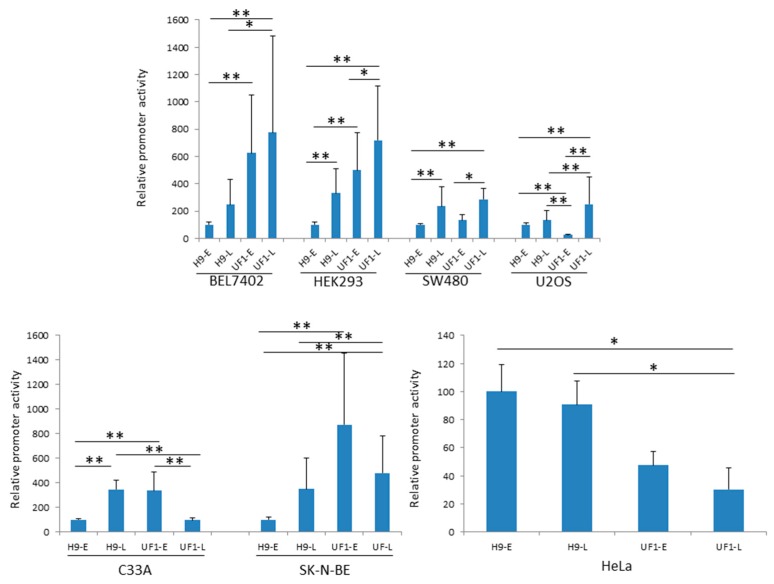
Comparison of the early and late promoter activity of HPyV9 and UF-1 in different human cell lines. Cells that were ~70% confluent were transfected with 400 ng of a reporter plasmid containing the *luciferase* gene driven by either the early or the late promoter of HPyV9 (respectively UF-1). Cells were harvest the next day and luciferase activity was measured and corrected for protein concentration in the sample. Each experiment was repeated three to five times, each time with three independent parallels and the results are the average of 9–15 values ± standard deviation. The activity of the HPyV9 early promoter (H9-E) was arbitrary set as 100. * *p* < 0.05; ** *p* < 0.01.

HPyV9 DNA can be found in urine, throat swabs, skin, and blood from healthy individuals [13,27,28,29,30,31], while viremia was observed in renal recipients and in patients with both kidney and pancreas transplantation [20]. However, little is known about the route of infection, cell tropism, transmission, and life cycle of HPyV9 and a permissive cell culture system is lacking. The effect of mutations in the NCCR on the properties of HPyV9 has not been investigated. In an effort to identify cell lines that may sustain the viral life cycle and to assess the effect of these differences on promoter activity, we compared the relative activities of the early and late HPyV9 promoter (hereafter referred to as H9-E and H9-L) with those of the early and late UF-1 promoter (UF1-E and UF1-L) in seven different human cell lines. The cell tropism of HPyV9 is not known, but because our previous study had shown that the HPyV9 promoter is relatively strong in BEL7402, HEK293, SK-N-BE, and SW480 cells, we selected these cells [12]. In addition, we chose one osteosarcoma cell line (U2OS), one human papillomavirus (HPV)-negative cervical cancer cell line (C33A), and the HPV18-positive cervical cancer cell line HeLa. The luciferase values that were obtained for all four promoters in HEK293 cells were 5–10× higher than the values for the corresponding promoters in C33A cells. The promoter activities in HeLa and U2OS were comparable, but up to 20-fold lower than in C33A cells. All four promoters had lowest activity in SK-N-BE, SW480, and BEL7402 cells (see Supplementary Materials). Differences in promoter strength may be partially explained by differences in transfection efficiency. Using an enhanced green fluorescent protein expression vector, it was estimated that all cells had a transfection efficiency of more than 75%, except for the BEL7402 (~50%) and C33A cells (~30%). For both HPyV9 and UF1, their late promoter was stronger (2- to 7-fold) than their early promoter in most of the cell lines that were tested. In HeLa and U2OS cells, the H9-E and H9-L promoters possessed similar activities and the same was true for the UF1-E and UF1-L promoters in BEL7402. In C33A cells, UF1-E was three-fold stronger than UF1-L (Appendix A). To compare the activity of all four promoters, we set the H9-E promoter activity arbitrarily as 100%. The UF1-E promoter was stronger as compared with the H9-E promoter in all cell lines, except in HeLa and U2OS cells where the basal HPyV9 promoter activity was, respectively, ~two- to four-fold higher (Figure 2). The H9-L promoter activity was higher than the H9-E promoter activity, except in U2OS (Figure 2) where there was no significant difference. For the late promoter activity, there was a tendency that the H9-L and UF1-L were comparable, except for C33A and HeLa cells, in which the H9-L promoter was on average approximately four-fold stronger, while UF1-L was stronger in BEL7402 cells (Figure 2). The UF1-E promoter was stronger than the UF1-L promoter in C33A, HeLa, and SK-N-BE cells, but weaker in HEK293, SW480, and U2OS cells (Figure 2). UF1-E and UF1-L promoter activity was comparable in BEL7402 cells (Figure 2).

### 2.2. The UF1 Late Promoter is more Potently Activated by HPyV9 LTAg than the HPyV9 Late Promoter

**Figure 3:**
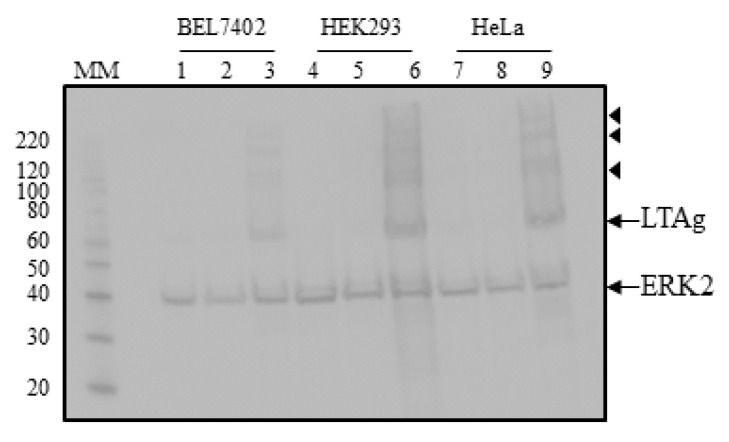
Detection of ectopic expressed HPyV9 LTAg in BEL7402, HEK293, and HeLa cells. Lanes 1–3: lysates of BEL7402 cells, lanes 4–6: lysates of HEK293 cells; lanes 7–9: lysates of HeLa cells. Lanes 1, 4, and 7: mock transfected cells; lanes 2, 5, and 8: cells transfected with empty vector pcDNA3.1(+); lanes 3, 6, and 9: cells transfected with expression plasmid for hemagglutinin (HA) tagged LTAg of HPyV9. Western blots were performed with anti-HA and anti-ERK2 antibodies. The molecular mass (in kDa) of the markers (lane MM) is indicated. The arrows indicate the bands corresponding to HPyV9, LTAg, and ERK2, respectively, while the bands marked with arrowheads may represent post-translationally modified HPyV9 LTAg.

Next, we examined the effect of HPyV9 LTAg on the early and late promoter activities of HPyV9 and UF-1. We decided to test in BEL7402, HEK293, and HeLa cells because some of the most obvious differences in early and/or late promoter activities between HPyV9 and UF-1 were observed in these cells. Cells were transfected with HPyV9 LTAg expression plasmid (the UF-1 LTAg amino acid sequence is completely identical to HPyV9) or with empty vector. Expression of HPyV9 LTAg in the three cell lines was confirmed by western blotting (Figure 3).

**Figure 4:**
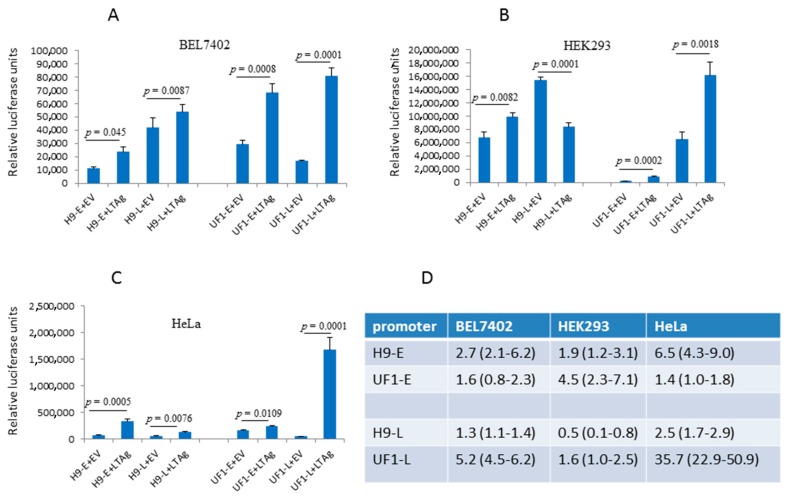
Effect of HPyV9 LTAg on the early and late promoters of HPyV9 and UF1. BEL7402 (panel **A**), HEK293 (panel **B**), and HeLa cells (panel **C**) were co-transfected with the luciferase reporter plasmid containing the H9-E, H9-L, UF1-E, or UF1-L promoter, and empty vector pcDNA3.1(+) or expression plasmid for HPyV9 LTAg. Luciferase values were measured and corrected for the protein concentration. Each experiment was repeated three to five times (Appendix A). A representative experiment for each promoter and each cell line is shown. Each bar represents the average of three independent parallels ± standard deviation. (Panel **D**) Fold induction of the promoter by HPyV9 LTAg. The average (range) of three to four independent experiments is given.

Cells were co-transfected with (i) the reporter plasmid containing the luciferase gene under control of the early or late promoter of, respectively, HPyV9 or UF-1, and (ii) empty expression vector pcDNA3.1(+) or HPyV9 LTAg vector. Dose-dependent transfection studies with increasing amounts of HPyV9 LTAg (0–1200 ng) or corresponding empty vector showed that co-transfection with 400 ng plasmid DNA/10^5^ cells was optimal (Ugo Moens, University of Tromsø, Norway, unpublished results, 2017). Higher concentrations hampered the HPyV9 promoters probably because the empty and HPyV9 LTAg expression vectors contain the strong cytomegalovirus promoter that competes out transcription factors required for the HPyV9 promoter. HPyV9 LTAg stimulated both H9-E and UF1-E promoters in all cell lines tested, although the luciferase expression levels differed (Figure 4A–C). H9-L and UF1-L promoters were also activated by HPyV9 LTAg, except in HEK293 cells were a 50% reduction was observed for H9-L (Figure 4B). Strongest activation was measured for the UF1-L promoter in HeLa cells (~36-fold increase; Figure 4C), while a 1.3- to 6.5-fold HPyV9 LTAg-mediated increase was observed for the HPyV9 and UF-1 promoters in the other experiments. No significant HPyV9 LTAg-mediated induction was observed for H9-L in BEL7402 cells (Figure 4A), while for all other promoters in all cell lines tested HPyV9 LTAg significantly affected promoter activity.

### 2.3. Sp1 Sites in the Late UF1 Promoter and HPyV9 LTAg

**Figure 5:**
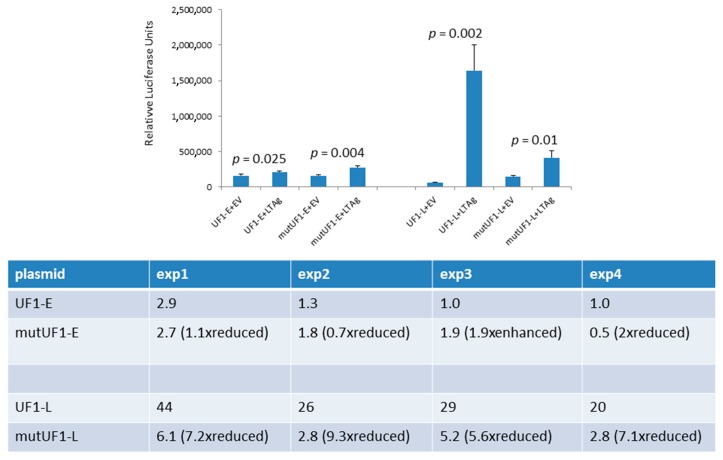
Effect of mutation of putative Sp1 motifs on HPyV9 LTAg-induced UF1-E and UF1-L promoter activity. HeLa cells were co-transfected with the luciferase reporter plasmid with wild-type UF1-E (respectively UF1-L) promoter or with mutated UF1-E (respectively mutated UF1-L) promoter and empty expression vector pcDNA3.1(+) (EV) or HPyV9 LTAg expression vector. Luciferase activity was measured and corrected for protein concentration. Top panel: a representative experiment is shown. The bars show the average of three parallels ± SD. Each experiment was performed 4 times (Appendix A). The table summarizes the promoter activity in the presence of HPyV9 LTAg (shown as fold induction) of the four experiments.

One of the differences between the UF1 and HPyV9 promoter is the presence of three Sp1 sites in the UF1 promoter, two of which are in the late promoter region (Figure 1). Previous studies had shown that SV40 LTAg can interact with Sp1 and stimulate transcription from Sp1 site-containing promoters [32,33]. Because Sp1 is constitutively expressed in HeLa cells [34] and the UF1-L promoter activity was strongest (on average 35-fold) stimulated by HPyV9 LTAg in these cells (Figure 4C,D), we examined the effect of HPyV9 LTAg on the activity of the UF1-E promoter and the UF1-L promoter in which the two distal Sp1 sites were mutated in HeLa cells. Destroying these two Sp1 motifs did not have an effect on the early promoter activity in the absence of HPyV9 LTAg, because the Sp1-mutated early promoter’s activity did not change (1.1 ± 0.4-fold; *n* = 4) when compared with that of the non-mutated promoter. However, the activity of the mutated late promoter was 2-fold (±0.3; *n* = 4) higher than the non-mutated promoter. Disrupting the two Sp1 sites reduced HPyV9 LTAg activation of the mutated late promoter 7.3-fold ± 1.5 (*n* = 4) as compared to the wild-type UF1 late promoter, while transactivation of the mutated UF1-E promoter was only slightly (1.1-fold ± 0.7) reduced (Figure 5).

## 3. Discussions

HPyV9 is one of the 14 polyomaviruses that have been detected in humans so far, but little is known about the biology of this virus. Antibodies against HPyV9 can be detected in ~20–50% of the adult population, while viral DNA is found with low frequency in urine, blood, skin, and throat swabs (reviewed in [31]). Attempts to propagate HPyV9 in cell culture have been unsuccessful [25,26]. In this study, we compared the early and late promoter activity of the originally described HPyV9 variant with that of the UF-1 isolate in seven different human cell lines. We had previously monitored the HPyV9 early and late promoter in ten different cell lines [12], and selected BEL7402, HEK293, SK-N-BE, and SW480 because their promoter activity was highest in these cells. In addition, we have also tested the human cervical cancer cell lines C33A and HeLa, and human osteosarcoma U2OS cells. The UF-1 early and late promoters were stronger than the HPyV9 promoters in most cell lines, except for HeLa cells where both UF-1 early and late promoter activity was lower than those of HPyV9, while the UF-1 early promoter was weaker in U2OS and the UF-1 late was weaker in C33A and SK-N-BE cells. The molecular basis for the difference in activity between the HPyV9 and UF-1 promoters in a specific cell line and between the different cells remains elusive, but the most obvious reason is the difference in the repertoire of transcription factors in each cell line.

Our study is the first to show that HPyV9 LTAg, in agreement with LTAg of other human polyomaviruses, influences early and late promoter activities. The amplitude of promoter activity stimulation by HPyV9 LTAg was different, with the late UF-1 promoter more inducible by HPyV9 LTAg, despite conservation of the putative LTAg-binding sites in the HPyV9 and UF-1 NCCR (Figure 1). The LTAg of polyomaviruses not only affects transcription by direct binding to these motifs in the promoter, but also through interaction with cellular transcription factors [1]. Hence, the differences in HPyV9 LTAg-induced promoter activity can be explained by distinct transcription factor binding sites. The UF-1 promoter possesses three putative Sp1 sites (5′-CCGCCC-3′), two of which are located in its late promoter region (Figure 1). The 5′-AGCTCATTTCATC-3′ deletion in HPyV9 does not seem to encompass putative binding sites using the PROMO transcription factor binding site prediction algorithm [35,36], while nucleotides spanning this region contain mutations that create putative binding motifs for STAT5A and Elk1. The possible contribution of these transcription factors in the regulation of HPyV9 promoter activity was not tested. Sp1 is known to be ubiquitously expressed at medium to high levels in all of the mammalian cells and tissues that have been tested [37,38]. LTAg of SV40 can interact with Sp1 and stimulate transcription of Sp1-containing promoters [32,33]. Accordingly, HPyV9 LTAg may more potently stimulate the UF1 promoter than the HPyV9 promoter through the additional Sp1 sites. However, the Sp1 family member Sp3 binds to the Sp1 motif with an affinity and specificity that are comparable with Sp1. Like Sp1, Sp3 is ubiquitously expressed, but in contrast to Sp1, Sp3 acts as a repressor [39,40]. Mutation of the two distal Sp1 sites did not affect the activity of the UF1-E promoter, but stimulated the UF1-L promoter in the absence of HPyV9 LTAg. However, in the presence of HPyV9 LTAg, induction of UF1-L was severely impaired. SV40 LTAg physically interacts with Sp1 and stimulates transcription of Sp1-containing promoters [32,33]. It is not known whether Sp3 can bind SV40 LTAg, but Sp1 and Sp3 share only ~40% amino acid identity. Whether HPyV9 LTAg binds Sp1 has to be proven, but supposing that HPyV9 LTAg does, the following scenario can be imagined. In the absence of HPyV9 LTAg, Sp1, and Sp3 may compete for the same motifs, and, depending on which of the proteins binds, stimulate (Sp1) or repress (Sp3) the promoter. HPyV9 LTAg, through its association with Sp1 may help to recruit Sp1 to the promoter, but not Sp3 assuming that Sp3 cannot interact with HPyV9 LTAg. Thus, intact Sp1 sites may bind Sp1 or Sp3, while HPyV9 LTAg may help to recruit Sp1 but not Sp3 to these sites. The deletion of the Sp1 site prevents Sp1 and Sp3 binding, but also LTAg-mediated recruitment of Sp1. The fact that the early and late promoter of HPyV9 was also induced by HPyV9 LTAg in HeLa cells suggests that other, Sp1-independent pathways, are operational. The UF1 promoter lacks a putative AML-1a (=RUNX1) site. This transcription factor is highly expressed in cells from myeloid and lymphoid tissue, while low transcript levels are found in HEK293, HeLa, U2OS, colon cancer, and neuroblastoma cells [37,38]. Because our study did not include myeloid and lymphoid cells, but rather cells with no or low AMP-1a expression levels, we did not investigate the role of this transcription factor. However, we cannot rule out a role of this transcription factor in HPyV9 promoter activity. Finally, LTAg stimulated the HPyV9 and UF-1 early and late promoters in all cell lines, except in HEK293 cells where the H9-L promoter was inhibited in the presence of LTAg. Further studies are required to unveil the mechanism of LTAg on the HPyV9 and UF-1 promoters in the different cell lines.

The natural host cells for HPyV9 remain unknown. In a previous study, we measured VP1 mRNA levels in Vero, RH, COS-7, HEK293 (all kidney-derived), BJAB (mouse hybridoma), HeLa, Huh-7 (hepatocellular carcinoma), and NIH3T3 (mouse fibroblast) cells transfected with complete HPyV9 genome [25]. VP1 mRNA levels increased over time, with highest copy number quantified in Vero cells and minimal increase in NIH3T3 and HeLa cells. Despite early (luciferase reporter assays in this study) and late viral promoter activity (this study and [25]), none of the cell lines were able to support a complete viral life cycle. These findings indicate that the presence of actively transcribed HPyV9 genome is not sufficient to generate infectious virus particles.

Western blot detection of ectopic expression of HPyV9 LTAg resulted in several bands corresponding to a molecular mass higher than the predicted 78 kDa. Toptan and colleagues, using a monoclonal antibody cross-reacting with the J-domain common in human polyomavirus LTAg and small T-antigen [41], detected bands corresponding to >78 kDa in HEK293 cells transfected with a HPyV9 LTAg expression plasmid [42]. These bands may represent post-translationally modified HPyV9 LTAg, as was shown for LTAg of other polyomaviruses [43,44,45].

In conclusion, the basal promoter activity of the HPyV9 variant UF-1, which was isolated from an AIDS patient, is stronger and more potently induced by LTAg in most of the cell lines we examined when compared with the original HPyV9 isolate. This suggests that the mutations in the UF1 NCCR may affect the life cycle of the virus. Further screening of specimens from healthy and diseased individuals is necessary to determine the host cell(s) for HPyV9, which may allow for establishing permissive cell cultures for virus propagation and unveiling a possible association with diseases.

## 4. Materials and Methods

### 4.1. Cell Lines

The human cell lines C33A (human papilloma virus negative cervical cancer), HEK293 (human embryonal kidney cells), HeLa (human papilloma virus positive cervical cancer), SK-N-BE (neuroblastoma), SW480 (colorectal adenocarcinoma), and U2OS (osteosarcoma) were maintained in Dulbecco’s Modified Eagle’s Medium (Sigma D5796, St. Louis, MO, USA), country) with 10% foetal bovine serum (Life Technologies, cat. No. 10500-064, Carlsbad, CA, USA). BEL-7402 (human hepatocellular carcinoma) was grown in DMEM with low glucose (Sigma D5546, St. Louis, MO, USA) [12]. All of the cells were kept at 37 °C in a humidified CO_2_ incubator.

### 4.2. Plasmids

The luciferase reporter plasmids containing the HPyV9 early or late promoter have been previously described [12]. The corresponding plasmids with the HPyV9 UF-1 early or late promoter were synthesized by GenScript as follows (Piscataway, NJ, USA). The complete NCCR was synthesized with cohesive *Hind*III at both ends and subsequently cloned in the *Hind*III site of pUC58. The NCCR was then excised and cloned in either orientation in the *Hind*III site of pGL3-basic. The empty expression vector pcDNA3.1(+) was purchased from Thermo Fisher Scientific (Waltham, MA, USA). The HPyV9 large T antigen expression vector was made by GenScript by cloning the synthesized cDNA into the *Kpn*I-*Apa*I sites of their pcDNA3.1+C-HA vector. Expressed HPyV9 LTAg has a C-terminal linked HA tag. The luciferase reporter plasmids with the UF1-E or UF1-L promoter with mutated Sp1 sites were generated by PCR-based site directed mutagenesis using primers mutSp1_UF1_fwd: 5′-GCTCATTTCATCAGCGCAGAGAAGAAGCCCGCCTTTTTTAAACGCGCCAATTTTGAACTTGG-3′ and mutSp1_UF1_rev: 5′-CCAAGTTCAAAATTGGCGCGTTTAAAAAAG GCGGGCTTCTTCTCTGCGCTGATGAAATGAGC-3′. These primers result in the mutation of the two Sp1 sites proximal to the late coding region (Figure 1). Mutations were confirmed by sequencing.

### 4.3. Transfection

Cells were seeded into twelve-well cell culture plates (Falcon cat. No. 353072, Corning Incorporated, Corning, NY, USA) and transfected the following day. Cells were approximately 70% confluent on the day of transfection. JetPrime (Polyplus-transfection, Illkirch, France) was used as transfection reagent. Cells were transfected with 400 ng luciferase plasmid according to the manufacturer’s instructions. In the experiments where the effect of HPyV9 LTAg on promoter activity was monitored, co-transfections were performed using 400 ng empty vector (pcDNA3.1(+)) or 400 ng HPyV9 LTAg expression plasmid.

### 4.4. Luciferase Assay

Cells were lysed 24 h post-transfection in 100 µL Luciferase Assay Tropix Lysis solution (Applied Biosystems, Foster City, CA, USA) with DTT added to a final concentration of 0.5 mM (Sigma, St. Louis, MO, USA) freshly added. Cells were scraped and transferred to Eppendorf tubes, followed by 3 min centrifugation at 12,000× *g* at room temperature in a Microfuge 22R centrifuge (Beckman Coulter, Bea, CA, USA). A 20 µL aliquot of each supernatant was subsequently transferred to 96-well microtiter plate and 50 µL luciferase buffer (Promega, Madison, WI, USA) was added. Light units were measured using CLARIOstar monochromator (520–620 nm) microplate reader (BMG Labtech GmbH, Ortenberg, Germany).

### 4.5. Protein Concentration Assay

The luciferase value of each sample was corrected relative to its protein concentration. Protein concentrations were determined using the Protein Quantification Assay from Macherey-Nagel (Düren, Germany) according to the manufacturer’s instructions with OD570 measured using the CLARIOstar monochromator microplate reader.

### 4.6. Western Blotting

Cells were plated out in 6-well plates and the next day transfected with 2 µg DNA (either pcDNA3.1 or pcDNA3.1-LTAg) using JetPrime. Twenty-four hours after transfection, the cells were washed briefly with phosphate-buffered saline (PBS, Biochrom GmbH) and harvested in NuPage LDS sample buffer (Invitrogen, Carlsbad, CA, USA) with 100 mM DTT. The samples were sonicated and heated for 10 min at 70 °C. Proteins were separated on NuPAGE™ Novex™ 4–12% Bis-Tris Protein Gels (Thermo Fisher Scientific, Waltham, MA, USA) and transferred onto a 0.45 μm PVDF Membrane (Merck Life Science AS, Darmstadt, Germany). The membrane was blocked in TBST (Tris-buffered saline with 0.1% Tween-20; Sigma, St. Louis, MO, USA) containing 5% (*w*/*v*) skim milk powder. Incubation with the primary anti-HA antibody (SC-805 Santa Cruz Biotechnology, Dallas TX, USA) or anti-ERK2 antibody (SC-154) was overnight at 4 °C in blocking buffer. Following three washes in TBST, the membrane was incubated with the polyclonal swine anti-rabbit secondary antibody that was conjugated with alkaline phosphatase (D0306, Dako, Santa Clara, CA, USA) solution for 1 h at room temperature. After four washes, detection and visualization were performed using CDP-Star chemiluminescent (C0712, Sigma, St. Louis, MO, USA) and the ImageQuant LAS 4000 imager (GE Healthcare, Pittsburgh, PA, USA). MagicMark™ XP Western Protein Standard (Thermo Fisher Scientific, Waltham, MA, USA) was used to estimate the molecular mass of the detected proteins.

### 4.7. Statistical Analysis

The *t*-test was employed to determine statistical differences between the promoter activities.

## Supplementary Materials

Supplementary materials can be found at www.mdpi.com/1422-0067/18/11/2414/s1.

## Abbreviations

EV	empty vector
HPyV9	human polyomavirus 9
H9-E	early promoter HPyV9
H9-L	late promoter HPyV9
LTAg	large T-antigen
NCCR	non-coding control region
PCR	Polymerase Chain Reaction
UF1-E	early promoter UF1
UF1-L	late promoter UF1

## Appendix A

Transfection results in seven human cell lines; effect of LTAg on HPyV9 and UF1 promoter in BEL7402, HEK293 and HeLa cells; effect of Sp1 mutation on LTAg-induced promoter activity.
