# Supplementary material for: A Role of Sp1 Binding Motifs in Basal and Large T-Antigen-Induced Promoter Activities of Human Polyomavirus HPyV9 and Its Variant UF-1
Source: Int J Mol Sci. 2017 Nov 14;18(11):2414. doi: 10.3390/ijms18112414 (PMC5713382; doi:10.3390/ijms18112414)
Supplement: Supplementary file 1 [file ijms-18-02414-s001.pdf]

Results transfection in seven human cell lines

Results transfections in seven human cell lines

Case Summaries

SKWe/l = SK cell+Wild type/H9+early/late promoter, SKVe = SK cell+Variant/UF1+early, HWe = HEK cell+Wild type/H9+eary, HVe = HEK cell+Variant/UF1+early, BELWe=BEL cell+Wild type/H9+early		ratio=luc/protein concentration	RelativeRatio=ratio/meanRatio x 100%	RelativeRatio2=ratio/meanRatio2 x 100%
BELHe	N	12	12	12
	Mean	11,3213	99,9827	99,9827
	Median	8,6175	101,9896	101,9896
	Std. Deviation	8,39754	18,68030	18,68030
	Minimum	1,70	63,45	63,45
	Maximum	26,93	131,26	131,26
BELHl	N	12	12	12
	Mean	22,6754	100,0335	246,4719
	Median	14,2473	99,1740	215,8608
	Std. Deviation	21,18865	11,30509	183,89991
	Minimum	4,14	85,96	51,06
	Maximum	62,07	116,52	532,31
BELUe	N	12	12	12
	Mean	51,8208	626,2552	626,2552
	Median	32,5267	529,0969	529,0969
	Std. Deviation	44,52618	421,88797	421,88797
	Minimum	17,75	155,56	155,56
	Maximum	153,98	1320,58	1320,58
BELUl	N	12	12	12
	Mean	48,7794	321,8155	774,7567
	Median	37,9151	292,5261	519,2039
	Std. Deviation	31,17919	165,12058	707,19811

	Minimum	18,32	153,21	153,21
	Maximum	105,19	560,77	1997,88
C33AHe	N	9	9	9
	Mean	964772,3469	100,0000	100,0000
	Median	689457,5893	103,1342	103,1342
	Std. Deviation	459878,81416	9,65341	9,65341
	Minimum	574882,35	85,88	85,88
	Maximum	1730706,47	111,82	111,82
C33AHl	N	9	9	9
	Mean	3070632,4128	100,0000	346,0499
	Median	2741740,5248	99,5685	389,3253
	Std. Deviation	817752,64344	12,17783	77,35476
	Minimum	2114814,81	83,77	221,96
	Maximum	4898584,91	122,79	422,66
C33AUe	N	9	9	9
	Mean	2839185,3606	335,7420	335,7420
	Median	3243407,7079	288,7011	288,7011
	Std. Deviation	822765,84585	154,09556	154,09556
	Minimum	1533044,44	171,07	171,07
	Maximum	3826086,96	588,50	588,50
C33AUl	N	9	9	9
	Mean	862054,7887	27,9527	96,4147
	Median	784569,7674	29,0800	101,4119
	Std. Deviation	227100,29157	2,49251	19,37709
	Minimum	592042,20	23,45	65,18
	Maximum	1219172,87	30,56	118,00
HEKHe	N	12	12	12
	Mean	763,6952	99,9853	99,9853

	Median	703,6146	98,9738	98,9738
	Std. Deviation	420,02811	18,82021	18,82021
	Minimum	271,88	57,51	57,51
	Maximum	1574,51	128,24	128,24
HEKHl	N	12	12	12
	Mean	3004,5692	100,1525	331,1995
	Median	2644,7232	98,4250	312,2218
	Std. Deviation	2245,26811	18,75196	179,73019
	Minimum	182,54	66,32	62,06
	Maximum	6709,38	132,48	556,84
HEKUe	N	12	12	12
	Mean	3078,7329	497,5071	497,5071
	Median	2822,7435	376,4573	376,4573
	Std. Deviation	856,12040	274,17135	274,17135
	Minimum	1927,39	270,94	270,94
	Maximum	4760,20	1120,26	1120,26
HEKUl	N	12	12	12
	Mean	4847,2184	486,2380	716,7112
	Median	4245,0400	138,3681	625,3852
	Std. Deviation	2605,29064	673,38174	401,46589
	Minimum	1984,69	80,69	326,62
	Maximum	10126,58	2065,16	1702,12
HELAHe	N	9	9	9
	Mean	59978,8837	99,9998	99,9998
	Median	33548,8127	101,2134	101,2134
	Std. Deviation	48372,99192	19,02066	19,02066
	Minimum	17741,74	67,26	67,26
	Maximum	145377,78	127,18	127,18

HELAHl	N	9	9	9
	Mean	54264,1857	100,0009	90,6030
	Median	33331,4917	100,0859	83,8070
	Std. Deviation	42713,96577	14,24418	16,90098
	Minimum	20145,95	74,30	75,74
	Maximum	127119,66	121,73	124,08
HELAUe	N	9	9	9
	Mean	28060,0776	47,4555	47,4555
	Median	15795,6731	48,9246	48,9246
	Std. Deviation	22949,41638	9,69537	9,69537
	Minimum	10876,75	34,04	34,04
	Maximum	73817,39	61,09	61,09
HELAUl	N	9	9	9
	Mean	19262,1154	35,2987	30,3175
	Median	12140,9922	37,9965	34,1043
	Std. Deviation	17759,12812	19,95866	15,20718
	Minimum	3345,61	10,44	10,64
	Maximum	44812,50	59,95	48,03
SKHe	N	12	12	12
	Mean	549,3852	100,0608	100,0608
	Median	467,9900	106,7869	106,7869
	Std. Deviation	426,65359	22,24295	22,24295
	Minimum	27,89	55,14	55,14
	Maximum	1307,48	123,70	123,70
SKHl	N	12	12	12
	Mean	1197,2878	100,0003	348,6782
	Median	739,3610	104,3274	304,4243
	Std. Deviation	1043,88636	15,40607	250,85098

	Minimum	194,91	73,49	43,74
	Maximum	2875,42	117,33	632,36
SKUe	N	12	12	12
	Mean	2955,8925	873,0944	873,0944
	Median	2915,8293	745,2803	745,2803
	Std. Deviation	1526,12410	579,12394	579,12394
	Minimum	715,07	285,58	285,58
	Maximum	4753,72	2101,13	2101,13
SKUl	N	12	12	12
	Mean	1921,0003	172,1900	477,7410
	Median	1733,5906	154,2552	374,2921
	Std. Deviation	1381,64219	69,66005	300,61632
	Minimum	205,47	77,47	122,49
	Maximum	4243,01	315,60	933,12
SWHe	N	15	15	15
	Mean	233,6555	100,1009	100,1009
	Median	201,7333	97,4895	97,4895
	Std. Deviation	180,05337	7,07023	7,07023
	Minimum	46,69	88,49	88,49
	Maximum	570,00	114,93	114,93
SWHl	N	15	15	15
	Mean	520,0577	99,9812	234,5721
	Median	567,2811	97,3756	260,6069
	Std. Deviation	354,54278	14,79878	142,80987
	Minimum	26,12	78,29	54,07
	Maximum	1056,12	130,77	505,63
SWUe	N	15	15	15
	Mean	306,1903	133,2945	133,2945

	Median	191,0065	125,9118	125,9118
	Std. Deviation	235,72088	40,27364	40,27364
	Minimum	42,34	65,49	65,49
	Maximum	680,60	201,95	201,95
SWUl	N	15	15	15
	Mean	619,6890	164,1420	285,7477
	Median	774,4186	122,7968	288,7577
	Std. Deviation	375,17461	98,21836	78,54655
	Minimum	88,92	76,30	153,32
	Maximum	1045,71	412,56	388,15
U2OSHe	N	9	9	9
	Mean	158350,3096	99,9999	99,9999
	Median	179724,4898	101,7363	101,7363
	Std. Deviation	64853,25435	12,83040	12,83040
	Minimum	67796,95	83,18	83,18
	Maximum	245196,58	117,61	117,61
U2OSHl	N	9	9	9
	Mean	169879,1378	100,0001	131,7283
	Median	160762,8866	97,3641	86,1692
	Std. Deviation	13369,24594	6,70875	73,24035
	Minimum	157404,41	88,15	76,35
	Maximum	192394,74	107,88	245,54
U2OSUe	N	9	9	9
	Mean	42872,0784	25,2747	25,2747
	Median	49425,0000	26,2590	26,2590
	Std. Deviation	22074,40424	5,80517	5,80517
	Minimum	11381,17	14,53	14,53
	Maximum	63174,83	30,91	30,91

U2OSUI	N	9	9	9
	Mean	288272,2078	168,2910	250,3346
	Median	269908,4967	164,0203	143,3998
	Std. Deviation	93686,71738	48,61384	200,91130
	Minimum	173606,56	104,25	83,27
	Maximum	413372,73	231,51	527,56
Total	N	312	312	312
	Mean	247643,8384	197,2779	283,6549
	Median	1891,0346	106,8862	126,5459
	Std. Deviation	734273,64045	282,84962	333,63965
	Minimum	1,70	10,44	10,64
	Maximum	4898584,91	2101,13	2101,13

Statistics on luciferase values in seven human cell lines

Statistics luciferase values in 7 cell lines

Cells	plasmid	X±SD	H9-E vs H9-L	UF1-E vs UF1-L	H9-E vs UF1-E	H9-E vs UF1-L	H9-L vs UF1-L	H9-L vs UF1-E
BEL7402	H9-E	11.321±8.398	0.0984	0.8481	**0.0053**	**0.0006**	**0.0254**	0.0527
	H9-L	22.675±21.189						
	UF1-E	51.821±44.526						
	UF1-L	48.780±31.179						
C33A	H9-E	964772±459879	**0.0001**	**0.0001**	**0.0001**	0.5564	**0.0001**	0.5578
	H9-L	3070632±817753						
	UF1-E	2839185±822766						
	UF1-L	862055±227100						
HEK293	H9-E	764±420	**0.0026**	**0.0360**	**0.0001**	**0.0001**	0.0769	0.9157
	H9-L	3004±2245						
	UF1-E	3079±856						
	UF1-L	4847±2605						
HeLa	H9-E	59979±48373	0.7939	0.3766	0.0926	**0.0307**	**0.0374**	0.1245
	H9-L	54264±42714						
	UF1-E	28060±22949						
	UF1-L	19262±17759						
SK-N	H9-E	549±426	0.0591	0.0956	**0.0001**	**0.0034**	0.1618	**0.0033**
	H9-L	1197±1044						
	UF1-E	2956±1526						
	UF1-L	1921±1382						
SW480	H9-E	234±180	**0.0094**	**0.0105**	0.3519	**0.0012**	0.4614	0.0617
	H9-L	520±355						
	UF1-E	306±236						

	UF1-L	620±375						
U2OS	H9-E	158351±64853	0.6086	**0.0001**	**0.0001**	**0.0035**	**0.0017**	**0.0001**
	H9-L	169879±13369						
	UF1-E	42872±22074						
	UF1-L	288272±93687						

Original data luciferase values transfections in seven human cell lines

name	cell	promoter	promoterregio	paralell	repeat	ratio	RelativeRatio
BELHe	BEL	H9	e	1	1	1.6961326	79.4069568
BELHe	BEL	H9	e	2	1	2.58802817	121.162367
BELHe	BEL	H9	e	3	1	2.11827957	99.1703918
BELHe	BEL	H9	e	1	2	12.2707424	105.237928
BELHe	BEL	H9	e	2	2	15.3051643	131.26213
BELHe	BEL	H9	e	3	2	7.39776952	63.4457077
BELHe	BEL	H9	e	1	4	8.5	104.808878
BELHe	BEL	H9	e	2	4	7.10309278	87.5843746
BELHe	BEL	H9	e	3	4	8.73509934	107.70776
BELHe	BEL	H9	e	1	5	26.9341317	115.20159
BELHe	BEL	H9	e	2	5	22.6708861	96.9670063
BELHe	BEL	H9	e	3	5	20.5362319	87.8367489
BELHl	BEL	H9	l	1	1	8.27748691	108.771181
BELHl	BEL	H9	l	2	1	6.73267327	88.4713964
BELHl	BEL	H9	l	3	1	7.82307692	102.79996
BELHl	BEL	H9	l	1	2	62.0670391	112.746665
BELHl	BEL	H9	l	2	2	47.32	85.9582198
BELHl	BEL	H9	l	3	2	55.7740586	101.315274
BELHl	BEL	H9	l	1	4	4.14102564	89.0543149
BELHl	BEL	H9	l	2	4	5.35148515	115.085702
BELHl	BEL	H9	l	3	4	4.47208122	96.1737896
BELHl	BEL	H9	l	1	5	22.6862745	97.0328251
BELHl	BEL	H9	l	2	5	27.2427184	116.521465
BELHl	BEL	H9	l	3	5	20.2170543	86.4715751
BELUe	BEL	UF1	e	1	1	17.7528736	831.127039
BELUe	BEL	UF1	e	2	1	24.7346939	1157.99129
BELUe	BEL	UF1	e	3	1	19.2653061	901.933807
BELUe	BEL	UF1	e	1	2	153.979058	1320.57511
BELUe	BEL	UF1	e	2	2	82.1452145	704.504413
BELUe	BEL	UF1	e	3	2	124.353448	1066.49613
BELUe	BEL	UF1	e	1	4	27.2797203	336.371397
BELUe	BEL	UF1	e	2	4	28.6842105	353.689402
BELUe	BEL	UF1	e	3	4	28.3034483	348.99443
BELUe	BEL	UF1	e	1	5	36.3692308	155.557018
BELUe	BEL	UF1	e	2	5	40.9007634	174.939108
BELUe	BEL	UF1	e	3	5	38.0821918	162.883626
BELUl	BEL	UF1	l	1	1	39.0239521	512.798319
BELUl	BEL	UF1	l	2	1	36.8062827	483.656803
BELUl	BEL	UF1	l	3	1	42.6746988	560.771338
BELUl	BEL	UF1	l	1	2	100.728477	182.976343
BELUl	BEL	UF1	l	2	2	105.191638	191.08381
BELUl	BEL	UF1	l	3	2	89.2405063	162.108095
BELUl	BEL	UF1	l	1	4	22.1445087	476.225993
BELUl	BEL	UF1	l	2	4	19.5571429	420.583717
BELUl	BEL	UF1	l	3	4	18.3195266	393.968315
BELUl	BEL	UF1	l	1	5	35.8195489	153.20594

BELUl	BEL	UF1	l	2	5	36.3763441	155.587443
BELUl	BEL	UF1	l	3	5	39.4700855	168.819869
C33AHe	C33A	H9	e	1	1	1608322.27	103.134208
C33AHe	C33A	H9	e	2	1	1730706.47	110.982135
C33AHe	C33A	H9	e	3	1	1339310.64	85.8837458
C33AHe	C33A	H9	e	1	2	655355.556	95.7097895
C33AHe	C33A	H9	e	2	2	633195.846	92.4735292
C33AHe	C33A	H9	e	3	2	765643.939	111.816585
C33AHe	C33A	H9	e	1	3	686076.459	105.527658
C33AHe	C33A	H9	e	2	3	689457.589	106.04772
C33AHe	C33A	H9	e	3	3	574882.353	88.4245297
C33AHl	C33A	H9	l	1	1	3608080.81	90.4427109
C33AHl	C33A	H9	l	2	1	3461400.36	86.7659148
C33AHl	C33A	H9	l	3	1	4898584.91	122.791401
C33AHl	C33A	H9	l	1	2	2741740.52	101.622426
C33AHl	C33A	H9	l	2	2	2686327.08	99.56853
C33AHl	C33A	H9	l	3	2	2665834.89	98.8089885
C33AHl	C33A	H9	l	1	3	2114814.81	83.7691751
C33AHl	C33A	H9	l	2	3	2711009.17	107.384817
C33AHl	C33A	H9	l	3	3	2747899.16	108.846053
C33AUe	C33A	UF1	e	1	1	3520153.06	225.731001
C33AUe	C33A	UF1	e	2	1	3466267.33	222.27556
C33AUe	C33A	UF1	e	3	1	2667816.96	171.074661
C33AUe	C33A	UF1	e	1	2	1976828.9	288.701112
C33AUe	C33A	UF1	e	2	2	1997093.19	291.660561
C33AUe	C33A	UF1	e	3	2	1533044.44	223.889703
C33AUe	C33A	UF1	e	1	3	3243407.71	498.879118
C33AUe	C33A	UF1	e	2	3	3826086.96	588.502913
C33AUe	C33A	UF1	e	3	3	3321969.7	510.962994
C33AUl	C33A	UF1	l	1	1	1196969.23	30.0040791
C33AUl	C33A	UF1	l	2	1	1016380.33	25.4773098
C33AUl	C33A	UF1	l	3	1	1219172.87	30.5606512
C33AUl	C33A	UF1	l	1	2	808005.571	29.9486714
C33AUl	C33A	UF1	l	2	2	784569.767	29.0800249
C33AUl	C33A	UF1	l	3	2	694400	25.7378887
C33AUl	C33A	UF1	l	1	3	742593.83	29.4146193
C33AUl	C33A	UF1	l	2	3	592042.202	23.4511724
C33AUl	C33A	UF1	l	3	3	704359.296	27.9001248
HEKHe	HEK	H9	e	1	1	271.883289	92.4270088
HEKHe	HEK	H9	e	2	1	320.637584	109.001082
HEKHe	HEK	H9	e	3	1	289.96328	98.5733207
HEKHe	HEK	H9	e	1	3	517.987805	85.2458372
HEKHe	HEK	H9	e	2	3	603.838384	99.3743637
HEKHe	HEK	H9	e	3	3	701.075269	115.376748
HEKHe	HEK	H9	e	1	4	1400.57307	114.072689
HEKHe	HEK	H9	e	2	4	1574.5098	128.239341
HEKHe	HEK	H9	e	3	4	706.153846	57.5142204

HEKHe	HEK	H9	e	1	5	1094.88	118.249074
HEKHe	HEK	H9	e	2	5	848.52	91.6417362
HEKHe	HEK	H9	e	3	5	834.32	90.1081099
HEKHl	HEK	H9	l	1	1	228.431877	94.2181384
HEKHl	HEK	H9	l	2	1	316.389549	130.496824
HEKHl	HEK	H9	l	3	1	182.544987	75.2918074
HEKHl	HEK	H9	l	1	3	1930.43478	103.150169
HEKHl	HEK	H9	l	2	3	1820.07366	97.2531721
HEKHl	HEK	H9	l	3	3	1863.93443	99.5968125
HEKHl	HEK	H9	l	1	4	5217.48401	103.020306
HEKHl	HEK	H9	l	2	4	3359.01163	66.3243827
HEKHl	HEK	H9	l	3	4	6709.375	132.478004
HEKHl	HEK	H9	l	1	5	5155.88	107.212027
HEKHl	HEK	H9	l	2	5	4608.16	95.8226677
HEKHl	HEK	H9	l	3	5	4663.11	96.965305
HEKUe	HEK	UF1	e	1	1	1927.38589	655.216852
HEKUe	HEK	UF1	e	2	1	3295.3668	1120.26339
HEKUe	HEK	UF1	e	3	1	2678.62267	910.600581
HEKUe	HEK	UF1	e	1	3	2686.74699	442.160981
HEKUe	HEK	UF1	e	2	3	3591.24088	591.014561
HEKUe	HEK	UF1	e	3	3	2219.15888	365.209479
HEKUe	HEK	UF1	e	1	4	4425.65598	360.45708
HEKUe	HEK	UF1	e	2	4	3369.98255	274.475484
HEKUe	HEK	UF1	e	3	4	4760.20408	387.70507
HEKUe	HEK	UF1	e	1	5	2958.74	319.549416
HEKUe	HEK	UF1	e	2	5	2508.67	270.94102
HEKUe	HEK	UF1	e	3	5	2523.02	272.490847
HEKUl	HEK	UF1	l	1	1	3066.09808	1264.63109
HEKUl	HEK	UF1	l	2	1	3252.74725	1341.6157
HEKUl	HEK	UF1	l	3	1	5006.96864	2065.15514
HEKUl	HEK	UF1	l	1	3	1984.689	106.049169
HEKUl	HEK	UF1	l	2	3	2430.45113	129.867865
HEKUl	HEK	UF1	l	3	3	2216.32653	118.426407
HEKUl	HEK	UF1	l	1	4	8182.82548	161.57159
HEKUl	HEK	UF1	l	2	4	10126.5823	199.951472
HEKUl	HEK	UF1	l	3	4	7438.17204	146.868253
HEKUl	HEK	UF1	l	1	5	5971.68	124.175877
HEKUl	HEK	UF1	l	2	5	3880.62	80.694108
HEKUl	HEK	UF1	l	3	5	4609.46	95.8497
HELAHe	HELA	H9	e	1	1	35290.3226	112.203747
HELAHe	HELA	H9	e	2	1	31156.5934	99.0607701
HELAHe	HELA	H9	e	3	1	27907.9755	88.7319581
HELAHe	HELA	H9	e	1	2	33548.8127	127.180002
HELAHe	HELA	H9	e	2	2	27847.0255	105.565129
HELAHe	HELA	H9	e	3	2	17741.7417	67.2570671
HELAHe	HELA	H9	e	1	3	123587.591	101.213365
HELAHe	HELA	H9	e	2	3	145377.778	119.058669

HELAHe	HELA	H9	e	3	3	97352.1127	79.7275422
HELAHl	HELA	H9	l	1	1	39025.0696	121.728905
HELAHl	HELA	H9	l	2	1	23820.6522	74.3025427
HELAHl	HELA	H9	l	3	1	33331.4917	103.969218
HELAHl	HELA	H9	l	1	2	22107.438	104.601079
HELAHl	HELA	H9	l	2	2	20145.9459	95.3203026
HELAHl	HELA	H9	l	3	2	21153.1532	100.085891
HELAHl	HELA	H9	l	1	3	95442.8571	87.084488
HELAHl	HELA	H9	l	2	3	127119.658	115.987206
HELAHl	HELA	H9	l	3	3	106231.405	96.9282331
HELAUe	HELA	UF1	e	1	1	15795.6731	50.2215219
HELAUe	HELA	UF1	e	2	1	15387.7551	48.9245679
HELAUe	HELA	UF1	e	3	1	11132.2751	35.3944904
HELAUe	HELA	UF1	e	1	2	16114.9425	61.0900433
HELAUe	HELA	UF1	e	2	2	13518.2927	51.2464183
HELAUe	HELA	UF1	e	3	2	10876.7507	41.2326119
HELAUe	HELA	UF1	e	1	3	73817.3913	60.4535332
HELAUe	HELA	UF1	e	2	3	54335.5263	44.4986539
HELAUe	HELA	UF1	e	3	3	41562.0915	34.0377144
HELAUl	HELA	UF1	l	1	1	3751.91816	11.7031665
HELAUl	HELA	UF1	l	2	1	3404.68227	10.6200514
HELAUl	HELA	UF1	l	3	1	3345.60907	10.4357873
HELAUl	HELA	UF1	l	1	2	12669.5157	59.945662
HELAUl	HELA	UF1	l	2	2	10887.931	51.5161156
HELAUl	HELA	UF1	l	3	2	12140.9922	57.4449594
HELAUl	HELA	UF1	l	1	3	40702.4793	37.1379764
HELAUl	HELA	UF1	l	2	3	41643.4109	37.9965062
HELAUl	HELA	UF1	l	3	3	44812.5	40.8880637
SKHe	SK	H9	e	1	2	27.887931	55.1362812
SKHe	SK	H9	e	2	2	61.2669683	121.128842
SKHe	SK	H9	e	3	2	62.5675676	123.700213
SKHe	SK	H9	e	1	3	1304.49102	115.356952
SKHe	SK	H9	e	2	3	780.511182	69.0210891
SKHe	SK	H9	e	3	3	1307.47664	115.620972
SKHe	SK	H9	e	1	4	422.916667	75.4844391
SKHe	SK	H9	e	2	4	607.894737	108.500319
SKHe	SK	H9	e	3	4	650	116.015493
SKHe	SK	H9	e	1	5	431.63	94.9242374
SKHe	SK	H9	e	2	5	458.2	100.767522
SKHe	SK	H9	e	3	5	477.78	105.073563
SKHl	SK	H9	l	1	2	194.90982	73.4926359
SKHl	SK	H9	l	2	2	309.494163	116.697773
SKHl	SK	H9	l	3	2	291.229236	109.810805
SKHl	SK	H9	l	1	3	677.744807	110.962001
SKHl	SK	H9	l	2	3	660.05291	108.065441
SKHl	SK	H9	l	3	3	494.578313	80.9735446
SKHl	SK	H9	l	1	4	800.977199	75.0519756

SKHl	SK	H9	l	2	4	1252.1978	117.331578
SKHl	SK	H9	l	3	4	1148.52941	107.617797
SKHl	SK	H9	l	1	5	2875.42	101.036927
SKHl	SK	H9	l	2	5	2793.75	98.1671943
SKHl	SK	H9	l	3	5	2868.57	100.79623
SKUe	SK	UF1	e	1	2	1062.75168	2101.13024
SKUe	SK	UF1	e	2	2	715.065913	1413.73253
SKUe	SK	UF1	e	3	2	757.08061	1496.79836
SKUe	SK	UF1	e	1	3	3479.86577	307.726694
SKUe	SK	UF1	e	2	3	3229.42643	285.580187
SKUe	SK	UF1	e	3	3	4388.57143	388.0841
SKUe	SK	UF1	e	1	4	2602.23214	464.460375
SKUe	SK	UF1	e	2	4	2587.6652	461.860388
SKUe	SK	UF1	e	3	4	2497.56098	445.778103
SKUe	SK	UF1	e	1	5	4730.99	1040.44116
SKUe	SK	UF1	e	2	5	4753.72	1045.43995
SKUe	SK	UF1	e	3	5	4665.78	1026.10015
SKUl	SK	UF1	l	1	2	262.598425	99.0152804
SKUl	SK	UF1	l	2	2	409.803922	154.520539
SKUl	SK	UF1	l	3	2	205.46875	77.4739829
SKUl	SK	UF1	l	1	3	1927.67123	315.602946
SKUl	SK	UF1	l	2	3	1385.14851	226.779829
SKUl	SK	UF1	l	3	3	1692.56198	277.110297
SKUl	SK	UF1	l	1	4	1774.61929	166.28274
SKUl	SK	UF1	l	2	4	1643.42629	153.989889
SKUl	SK	UF1	l	3	4	1918.13472	179.73021
SKUl	SK	UF1	l	1	5	4243.01	149.091503
SKUl	SK	UF1	l	2	5	3503.62	123.11071
SKUl	SK	UF1	l	3	5	4085.94	143.572355
SWHe	SW	H9	e	1	1	201.733333	96.5832017
SWHe	SW	H9	e	2	1	240.063291	114.934309
SWHe	SW	H9	e	3	1	184.819277	88.4853148
SWHe	SW	H9	e	1	2	87.3700306	92.3768562
SWHe	SW	H9	e	2	2	104.537572	110.528201
SWHe	SW	H9	e	3	2	91.8154762	97.0770524
SWHe	SW	H9	e	1	3	49.6978852	102.894172
SWHe	SW	H9	e	2	3	46.6927083	96.672274
SWHe	SW	H9	e	3	3	48.5100287	100.434842
SWHe	SW	H9	e	1	4	523.193717	96.7909345
SWHe	SW	H9	e	2	4	526.969697	97.4894914
SWHe	SW	H9	e	3	4	570	105.450105
SWHe	SW	H9	e	1	5	292.86	106.560419
SWHe	SW	H9	e	2	5	279.76	101.793836
SWHe	SW	H9	e	3	5	256.81	93.4432194
SWHl	SW	H9	l	1	1	1056.11511	114.477818
SWHl	SW	H9	l	2	1	875.531915	94.9034648
SWHl	SW	H9	l	3	1	836	90.6183947

SWHl	SW	H9	l	1	2	246.481994	101.663021
SWHl	SW	H9	l	2	2	286.646707	118.229205
SWHl	SW	H9	l	3	2	193.610451	79.8558265
SWHl	SW	H9	l	1	3	43.6239782	130.767321
SWHl	SW	H9	l	2	3	30.3286385	90.9131849
SWHl	SW	H9	l	3	3	26.118068	78.2915707
SWHl	SW	H9	l	1	4	503.921569	86.4997457
SWHl	SW	H9	l	2	4	567.281106	97.3756125
SWHl	SW	H9	l	3	4	676.506024	116.124418
SWHl	SW	H9	l	1	5	780.43	95.224325
SWHl	SW	H9	l	2	5	863.59	105.371109
SWHl	SW	H9	l	3	5	814.68	99.4033457
SWUe	SW	UF1	e	1	1	201.698113	96.5663394
SWUe	SW	UF1	e	2	1	189.558824	90.7544518
SWUe	SW	UF1	e	3	1	136.789668	65.4903375
SWUe	SW	UF1	e	1	2	191.006494	201.952309
SWUe	SW	UF1	e	2	2	160.027322	169.197846
SWUe	SW	UF1	e	3	2	168.516484	178.173486
SWUe	SW	UF1	e	1	3	61.7473684	127.841342
SWUe	SW	UF1	e	2	3	42.3364486	87.6531027
SWUe	SW	UF1	e	3	3	53.216	110.178054
SWUe	SW	UF1	e	1	4	671.484375	124.224734
SWUe	SW	UF1	e	2	4	680.603448	125.911764
SWUe	SW	UF1	e	3	4	667	123.395123
SWUe	SW	UF1	e	1	5	520	189.207874
SWUe	SW	UF1	e	2	5	435.47	158.450679
SWUe	SW	UF1	e	3	5	413.4	150.42026
SWUl	SW	UF1	l	1	1	774.418605	83.9432665
SWUl	SW	UF1	l	2	1	703.921569	76.3017255
SWUl	SW	UF1	l	3	1	810.731707	87.8794328
SWUl	SW	UF1	l	1	2	273.10705	112.644689
SWUl	SW	UF1	l	2	2	269.59799	111.197356
SWUl	SW	UF1	l	3	2	297.720798	122.796782
SWUl	SW	UF1	l	1	3	137.630522	412.561517
SWUl	SW	UF1	l	2	3	111.20462	333.347184
SWUl	SW	UF1	l	3	3	88.9151874	266.532336
SWUl	SW	UF1	l	1	4	1027.09677	176.30444
SWUl	SW	UF1	l	2	4	1041.34078	178.749469
SWUl	SW	UF1	l	3	4	828.75	142.257583
SWUl	SW	UF1	l	1	5	999.77	121.98714
SWUl	SW	UF1	l	2	5	885.42	108.034701
SWUl	SW	UF1	l	3	5	1045.71	127.592518
U2OSHe	U2OS	H9	e	1	1	216618.557	115.087348
U2OSHe	U2OS	H9	e	2	1	156555.556	83.1764551
U2OSHe	U2OS	H9	e	3	1	191489.13	101.736326
U2OSHe	U2OS	H9	e	1	2	245196.581	117.614381
U2OSHe	U2OS	H9	e	2	2	200504.505	96.176762

U2OSHe	U2OS	H9	e	3	2	179724.49	86.2091329
U2OSHe	U2OS	H9	e	1	3	85841.1765	109.554178
U2OSHe	U2OS	H9	e	2	3	81425.8373	103.919134
U2OSHe	U2OS	H9	e	3	3	67796.9543	86.5253708
U2OSHl	U2OS	H9	l	1	1	160220.43	97.3641088
U2OSHl	U2OS	H9	l	2	1	159465.241	96.9051888
U2OSHl	U2OS	H9	l	3	1	173989.529	105.731431
U2OSHl	U2OS	H9	l	1	2	179641.304	107.877125
U2OSHl	U2OS	H9	l	2	2	160762.887	96.5403705
U2OSHl	U2OS	H9	l	3	2	159168.317	95.582809
U2OSHl	U2OS	H9	l	1	3	185865.385	104.094192
U2OSHl	U2OS	H9	l	2	3	157404.412	88.1545808
U2OSHl	U2OS	H9	l	3	3	192394.737	107.750966
U2OSUe	U2OS	UF1	e	1	1	58181.8182	30.9114382
U2OSUe	U2OS	UF1	e	2	1	48105.2632	25.5578619
U2OSUe	U2OS	UF1	e	3	1	49425	26.2590253
U2OSUe	U2OS	UF1	e	1	2	63174.8252	30.3033098
U2OSUe	U2OS	UF1	e	2	2	63078.7402	30.2572204
U2OSUe	U2OS	UF1	e	3	2	60756.3025	29.1432078
U2OSUe	U2OS	UF1	e	1	3	17207.5472	21.9610072
U2OSUe	U2OS	UF1	e	2	3	11381.1659	14.5251304
U2OSUe	U2OS	UF1	e	3	3	14538.0435	18.5540725
U2OSUl	U2OS	UF1	l	1	1	278218.391	169.07011
U2OSUl	U2OS	UF1	l	2	1	252579.235	153.48949
U2OSUl	U2OS	UF1	l	3	1	269908.497	164.020283
U2OSUl	U2OS	UF1	l	1	2	203801.724	122.385797
U2OSUl	U2OS	UF1	l	2	2	202137.931	121.386666
U2OSUl	U2OS	UF1	l	3	2	173606.557	104.253175
U2OSUl	U2OS	UF1	l	1	3	401345.133	224.773954
U2OSUl	U2OS	UF1	l	2	3	413372.727	231.510026
U2OSUl	U2OS	UF1	l	3	3	399479.675	223.729201

Effect of HPyV9 LTAg on HPyV9 and UF-1 promoters in BEL7402, HEK293 and HeLa cells

EFFECT OF HPyV9 LTAg ON THE H9 AND UF1 EARLY AND LATE PROMOTERS IN BEL7401, HEK293 AND HeLa CELLS

Effect HPyV9 LTAg on **H9-E** and **UF1-E** promoters in **BEL7401** cells

plasmid	LUC	Average LUC+SD	protein	LUC/prot	Average+SD
H9-E+EV	799<br>761<br>817	792+29	0,291<br>0,252<br>0,333	2746<br>3020<br>2453	2740+284
H9-E+LT	2612<br>2503<br>2567	2561+55	0,267<br>0,248<br>0,251	9783<br>10093<br>10227	10034+228
p (EV versus LT)		p=0.0001			p=0.0001 (3.7x)
UF1-E+EV	6959<br>7350<br>6375	6895+491	0,217<br>0,215<br>0,237	32069<br>34186<br>26899	31051+3749
UF1-E+LT	8068<br>8463<br>7297	7943+593	0,237<br>0,339<br>0,272	34042<br>24965<br>26827	28611+4794 (0.9x)
p (EV versus LT)		p=0.0778			p=0.5256

plasmid	LUC	protein	LUC/prot	Average±SD
H9-E+EV	1662<br>1640<br>1795	0,186<br>0,176<br>0,195	8935<br>9318<br>9205	9153±197
H9-E+LT	6221<br>7261<br>5334	0,222<br>0,244<br>0,216	28023<br>29758<br>24694	27492±2573 (3.0x)
p (EV versus LT)				p=0.0003
UF1-E+EV	9302<br>10143<br>9013	0,194<br>0,219<br>0,208	47948<br>46315<br>43332	45865±2341
UF1-E+LT	8738<br>8320<br>6895	0,224<br>0,205<br>0,185	39009<br>40585<br>37270	38965±1658 (0.8x)
p (EV versus LT)				p=0.0140

plasmid	LUC	prot	LUC/prot	Average+SD
H9-E+EV	3696<br>3504<br>3317	0,219<br>0,214<br>0,186	16877<br>16374<br>17833	17028+741
H9-E+LTAg	7670<br>8426<br>9209	0,230<br>0,226<br>0,245	33348<br>37283<br>37588	36073+2365
LTAg				P=0.0002 (2.1x)
UF1-E+EV	8567<br>6943<br>8488	0,275<br>0,235<br>0,244	31153<br>29545<br>34787	31828+2685
UF1-E+LTAg	13117<br>12457<br>12981	0,237<br>0,219<br>0,219	55346<br>56881<br>59274	57167+1980
LTAg				P=0.0002 (1.8x)

plasmid	LUC	protein	LUC/prot	Average±SD
H9-E+pcDNA3.1	2383<br>2160<br>2339	0,197<br>0,219<br>0,193	12096<br>9863<br>12119	11359±1296
H9-E+LTAg	5246<br>5896<br>5964	0,263<br>0,220<br>0,238	19947<br>26800<br>25059	23935±3562 (2.1x)
EV vs LTAg				p=0.0045
UF1-E+pcDNA3.1	6100<br>6933<br>8884	0,235<br>0,231<br>0,279	25957<br>30013<br>31842	29271±3012
UF1-E+LTAg	11572<br>12888<br>12921	0,191<br>0,184<br>0,175	60586<br>70043<br>73834	68154±6823 (2.3x)
EV vs LTAg				p=0.0008

plasmid	LUC	protein	LUC/protein	Average±SD
UF1-E+EV	6605<br>5892<br>6689	0,198<br>0,213<br>0,221	33359<br>27662<br>30267	30429±2852
UF1-E+LTAg	12239<br>12319<br>10133	0,180<br>0,210<br>0,171	67994<br>58662<br>59257	61971±5225 (2.0x)
EV vs. LTAg				p=0.0008

Effect HPyV9 LTAg on **H9-L** and **UF1-L** promoters in **BEL7401** cells

plasmid	LUC	Protein conc	LUC/prot	Average±SD
H9-L+pcDNA3.1	8917<br>7637<br>10164	0,198<br>0,232<br>0,213	45035<br>32918<br>47718	41890±7885
H9-L+LTAg	14912<br>11914<br>12285	0,249<br>0,238<br>0,234	59888<br>50059<br>52500	54149±5118
pcDNA3.1 vs LTAg				0.0868 (1.3x)
UF1-L+pcDNA3.1	4154<br>3755<br>3808	0,258<br>0,209<br>0,235	16101<br>17967<br>16204	16757±1049
UF1-L+LTAg	19776<br>20699<br>16706	0,227<br>0,255<br>0,223	87119<br>81173<br>74915	81069±6103 (4.8x)
pcDNA3.1 vs LTAg				0.0001

plasmid	LUC	protein	LUC/prot	Average±SD
H9-L+pcDNA3.1	13875<br>14715<br>12931	0,193<br>0,188<br>0,191	71891<br>78271<br>67702	72621±5322
H9-L+LTag	23074<br>25864<br>23067	0,242<br>0,256<br>0,234	95347<br>101031<br>98577	98318±2851
pcDNA3.1 vs LTAg				0.0018 (1.4x)
UF1-L+pcDNA3.1	6209<br>6112<br>5356	0,228<br>0,222<br>0,271	27232<br>27532<br>19764	24843±4401
UF1-L+LTAg	46358<br>37143<br>35616	0,273<br>0,263<br>0,240	169810<br>141228<br>148400	153146±14870 (6.2x)
pcDNA3.1 vs LTAg				0.0001

plasmid	LUC	protein	LUC/prot	Average+SD
H9-L+EV	8284<br>8397<br>9557	0,126<br>0,115<br>0,140	65746<br>73017<br>68264	69009+3692
H9-L+LTAg	14969<br>14864<br>14866	0,175<br>0,157<br>0,152	85537<br>94675<br>97803	92672+6374 (1.3x)
EV vs LTAg				p=0.0051
UF-1+EV	3253<br>3952<br>2932	0,141<br>0,146<br>0,128	23071<br>27068<br>22906	24348+2357
UF1-L+LTag	15757<br>16353<br>13807	0,155<br>0,139<br>0,130	101658<br>117647<br>106208	108504+8238 (4.5x)
EV vs LTAg				p=0.0001

Effect HPyV9 LTAg on **H9-E** and **UF1-E** promoters in **HEK293** cells

plasmid	LUC	Protein conc	LUC/prot	Average±SD
H9-E+EV	3685000<br>4269000<br>4363000	0,243<br>0,238<br>0,297	15164609<br>17936975<br>14690236	15930607±1753680
H9-E+LTAg	6110000<br>6890000<br>7210000	0,313<br>0,339<br>0,455	19520767<br>20324484<br>15846154	18563802±2387613
LTAg				P=0.1985 (1.2x)
UF1-E+EV	15840<br>11846<br>10693	1,011<br>0,856<br>0,660	15668<br>13839<br>16202	15236±1239
UF1-E-LTAg	52761<br>39640<br>45277	0,907<br>0,390<br>0,793	58171<br>101641<br>57096	72303±25413
LTAg				P=0.0178 (4.7x)

plasmid	LUC	prot	LUC/prot	Average±SD
H9-E+EV	40622<br>57923<br>72115	0,638<br>0,616<br>0,680	63671<br>94031<br>106051	87918±21841
H9-E+LTAg	136159<br>148043<br>173293	0,531<br>0,558<br>0,583	256420<br>265310<br>297244	272991±21469
LTAg				P=0.0005 (3.1x)
UF1-E+EV	3768<br>3302<br>6040	0,737<br>0,766<br>0,787	5113<br>4311<br>7675	5700±1757
UF1-E-LTAg	45766<br>34547<br>23706	0,880<br>0,878<br>0,773	52007<br>39347<br>30668	40674±10731
LTAg				P=0.0051 (7.1x)

plasmid	LUC	prot	LUC/prot	Average+SD
H9-E+EV	2799000<br>3166000<br>2841000	0,445<br>0,405<br>0,456	6289888<br>7817284<br>6230263	6779145+899549
H9-E+LTAg	4278000<br>3805000<br>4510000	0,444<br>0,404<br>0,424	9635135<br>9418317<br>10636792	9896748+650001
LTAg				P=0.0082 (1.5x)
UF1-E+EV	8172<br>9203<br>7629	0,509<br>0,465<br>0,484	16055<br>19791<br>15762	17203+2246
UF1-E+LTAg	15993<br>21852<br>18581	0,440<br>0,503<br>0,477	36348<br>43443<br>38954	39582+3589
LTAg				P=0.0008 (2.3x)

plasmid	LUC	prot	LUC/prot	Average+SD
H9-E+EV	2704000<br>2557000<br>2512000	0,284<br>0,309<br>0,287	9521127<br>8275081<br>8752613	8849607+628660
H9-E+LTAg	5160000<br>5519000<br>5161000	0,326<br>0,364<br>0,333	15828221<br>15162088<br>15498499	15496269+333072
LTAg				P=0.0001 (1.8x)
UF1-E+EV	75256<br>99781<br>92939	0,378<br>0,371<br>0,387	199090<br>268951<br>240152	236064+35109
UF1-E+LTAg	328587<br>302556<br>336606	0,333<br>0,365<br>0,346	986748<br>828921<br>972850	929506+87386
LTAg				P=0.0002 (3.9x)

Effect HPyV9 LTAg on **H9-L** and **UF1-L** promoters in **HEK293** cells

plasmid	LUC	Prot. Conc.	LUC/port	Average±SD
H9-L+EV	57474<br>80457<br>135893	0,972<br>0,893<br>1,467	10354595<br>9845039<br>7862069	9353901±1316846
H9-L+LTAg	1565000<br>2114000<br>2233000	0,917<br>1,012<br>1,079	5439349<br>4706970<br>4984448	5043589±369754
LTAg				P=0.0055 (0.1x)
UF1-L+EV	1318000<br>716263<br>811090	2,017<br>0,949<br>1,170	653446<br>754756<br>693239	700480±51042
UF1-L+LTAg	282865<br>826461<br>602388	0,380<br>1,010<br>1,255	744382<br>818278<br>479990	680883±177858
LTAg				P=0.8634 (1.0x)

plasmid	LUC	Prot. Conc.	LUC/port	Average±SD
H9-L+EV	42771000<br>40811000<br>44872000	0,725<br>0,739<br>0,859	58994483<br>55224628<br>52237485	55485532±3386046
H9-L+LTAg	24416000<br>24652000<br>23099000	0,960<br>0,810<br>0,788	25433333<br>30434568<br>29313452	28393784±2624391
LTAg				P=0.0004 (0.5x)
UF1-L+EV	331057<br>453039<br>479801	0,749<br>0,940<br>0,876	441999<br>481956<br>547718	490558±53382
UF1-L+LTAg	957553<br>615229<br>559230	0,833<br>0,750<br>0,710	1149523<br>820305<br>787648	919159±200168
LTAg				P=0.0231 (1.9x)

plasmid	LUC	prot	LUC/prot	Average±SD
H9-L+EV	8096000<br>7552000<br>7572000	0,341<br>0,336<br>0,328	23741935<br>22476190<br>23085366	23101164±633020
H9-L+LTAg	5657000<br>6095000<br>6077000	0,263<br>0,301<br>0,363	21509506<br>20249169<br>16741047	19499907±2470950
LTAg				P=0.0708 (0.8x)
UF1-L+EV	29573000<br>23560000<br>28352000	0,820<br>0,757<br>0,846	36064634<br>31122853<br>33513002	33566830±2471330
UF1-L+LTAg	22872000<br>21653000<br>21063000	0,775<br>0,852<br>0,865	29512258<br>25414319<br>24350289	26425622±2725533
LTAg				P=0.0283 (0.8x)

plasmid	LUC	protein	LUC/prot	Average±SD
H9-L+EV	3710786<br>3108013<br>3241965	0,247<br>0,204<br>0,203	15023425<br>15235358<br>15970271	15409685±496912
H9-L+LTAg	2290525<br>2034182<br>2172750	0,297<br>0,244<br>0,239	7712205<br>8336811<br>9091004	8380007±690414
LTAg				P=0.0001 (0.5x)
UF1-L+EV	1619123<br>1314889<br>1560641	0,247<br>0,241<br>0,203	6555154<br>5455971<br>7687887	6566337±1116000
UF1-L+LTAg	4427185<br>4523559<br>5512879	0,288<br>0,306<br>0,299	15372170<br>14782873<br>18437722	16197588±1962261
LTAg				P=0.0018 (2.5x)

Effect HPyV9 LTAg on **H9-E** and **UF1-E** promoters in **HeLa** cells

plasmid	LUC	Prot conc	LUC/prot	Average±SD
H9-E+EV	23519<br>23289<br>26209	0,283<br>0,351<br>0,292	83106<br>66350<br>89757	79738±12062
H9-E+LTAg	182138<br>169001<br>165576	0,391<br>0,399<br>0,338	465826<br>423561<br>489870	459752±33569
LTAg				P=0.0001 (5.8x)
UF1-E+EV	40718<br>41497<br>57559	0,390<br>0,422<br>0,470	104405<br>98334<br>122466	108402±12553
UF1-E+LTAg	73832<br>66129<br>113069	0,390<br>0,531<br>0,523	185508<br>169562<br>212936	189335±21939
LTAg				P=0.0052 (1.8x)

plasmid	LUC	Prot conc	LUC/prot	Average±SD
H9-E+EV	14288<br>12888<br>12064	0,250<br>0,191<br>0,186	57152<br>67476<br>64860	63163±5367
H9-E+LTAg	47580<br>56000<br>53976	0,173<br>0,221<br>0,187	275029<br>253394<br>288642	272355±17776
LTAg				P=0.0001 (4.3x)
UF1-E+EV	20553<br>22008<br>22896	0,488<br>0,645<br>0,553	42117<br>34121<br>41403	39214±4425
UF1-E+LTAg	21307<br>22625<br>19026	0,523<br>0,475<br>0,507	40740<br>47632<br>37527	41966±5163
LTAg				P=0.5218 (1.1x)

plasmid	LUC	prot	LUC/prot	Average+SD
H9-E+EV	23610<br>23332<br>20222	0,238<br>0,233<br>0,226	99202<br>100137<br>89478	96272+5903
H9-E+LTAg	212182<br>215822<br>185888	0,247<br>0,236<br>0,245	859036<br>914500<br>758727	844088+78955
LTAg				P=0.0001 (8.8x)
UF1-E+EV	22771<br>25635<br>24598	0,244<br>0,248<br>0,274	93324<br>103367<br>89774	95488+7050
UF1-E+LTAg	39880<br>40393<br>41437	0,275<br>0,299<br>0,243	145018<br>135094<br>170523	150212+18277
LTAg				P=0.0084 (1.6x)

plasmid	LUC	prot	LUC/prot	Average+SD
H9-E+EV	9318<br>8785<br>8629	0,137<br>0,122<br>0,133	68015<br>72008<br>64880	68301+3573
H9-E+LTAg	61630<br>47755<br>46710	0,162<br>0,160<br>0,149	380432<br>298469<br>313490	330797+43636
LTAg				P=0.0005 (4.8x)
UF1-E+EV	44784<br>45394<br>42253	0,250<br>0,282<br>0,295	179136<br>160972<br>143231	161113+17953
UF1-E+LTAg	50968<br>68687<br>57045	0,246<br>0,283<br>0,243	207187<br>242710<br>234753	228217+18642
LTAg				P=0.0109 (1.4x)

plasmid	LUC	Prot	LUC/prot	Average+SD
H9-E+EV	12279<br>13077<br>9742	0,190<br>0,173<br>0,148	64626<br>75590<br>65824	68680+6014
H9-E+LTAg	147342<br>104549<br>100081	0,183<br>0,241<br>0,161	805148<br>433813<br>621621	620194+185672
LTAg				P=0.0068 (9.0x)
UF1-E	4206<br>4655<br>4129	0,198<br>0,214<br>0,221	21242<br>21752<br>18683	20559+1645
UF1-E+LTAg	38814<br>45311<br>41244	0,197<br>0,190<br>0,157	197025<br>238479<br>262701	232735+33213
LTAg				P=0.7160 (1.0x)

Effect HPyV9 LTAg on **H9-L** and **UF1-L** promoters in **HeLa** cells

plasmid	LUC	Prot conc	LUC/prot	Average±SD
H9-L+EV	49243<br>61564<br>73793	0,388<br>0,441<br>0,443	126915<br>139601<br>166576	144364±20255
H9-L+LTAg	113079<br>159329<br>148592	0,257<br>0,343<br>0,443	439996<br>464516<br>335422	413311±68559
LTAg				P=0.0029 (2.9x)
UF1-L+EV	30553<br>33971<br>33450	0,433<br>0,545<br>0,423	70561<br>62332<br>79078	70657±8373
UF1-L+LTAg	525728<br>709223<br>597054	0,341<br>0,436<br>0,356	1541724<br>1626658<br>1677118	1615167±68425
LTAg				P=0.0001 (22.9x)

plasmid	LUC	Prot conc	LUC/prot	Average±SD
H9-L+EV	4722<br>4100<br>4450	0,440<br>0,400<br>0,503	10732<br>10250<br>8847	9943±979
H9-L+LTAg	117883<br>165269<br>119700	0,625<br>0,650<br>0,360	188613<br>254260<br>332500	258458±72035
LTAg				P=0.0039 (2.9x)
UF1-L+EV	13929<br>7896<br>7770	0,151<br>0,145<br>0,194	92245<br>54455<br>40052	62251±26956
UF1-L+LTAg	575011<br>621756<br>729905	0,179<br>0,152<br>0,331	3212352<br>4090500<br>2205151	3169334±943410
LTAg				P=0.0047 (50.9x)

plasmid	LUC	prot	LUC/prot	Average+SD
H9-L+EV	11128<br>7175<br>9897	0,103<br>0,084<br>0,088	108039<br>85417<br>112466	101974+14509
H9-L+LTAg	26093<br>15124<br>17259	0,121<br>0,113<br>0,097	215645<br>133841<br>177928	175805+40943
LTAg				P=0.0422 (1.7x)
UF1-L+EV	8674<br>6907<br>7831	0,181<br>0,220<br>0,155	47923<br>31395<br>50523	43280+10375
UF1-L+LTAg	166997<br>288318<br>212557	0,169<br>0,177<br>0,158	988148<br>1628915<br>1345297	1320787+321086
LTAg				P=0.0023 (31x)

plasmid	LUC	protein	LUC/prot	Average+SD
H9-L+EV	5293<br>4226<br>4048	0,088<br>0,082<br>0,079	60148<br>51537<br>51241	54309+5059
H9-L+LTAg	15106<br>10482<br>14818	0,145<br>0,090<br>0,099	104179<br>116467<br>149677	123441+23537
LTAg				P=0.0076 (2.3x)
UF1-L+EV	3682<br>3936<br>3765	0,094<br>0,085<br>0,083	39170<br>46306<br>45361	43612+3876
UF1-L+LTAg	172326<br>170863<br>217762	0,116<br>0,108<br>0,112	1485569<br>1582065<br>1944304	1670646+241856
LTAg				P=0.0003 (38x)

Effect of mutation of the Sp1 sites

Transfection 06.09.2017: UF1-E and mutUF1-E in HeLa

plasmid	LUC	prot	LUC/prot	Average±SD
UF1-E+EV	16250<br>12795<br>16261	0,223<br>0,202<br>0,224	72870<br>63342<br>72594	69602±5423
UF1-E+LTAg	39201<br>34944<br>39076	0,200<br>0,165<br>0,194	196005<br>211782<br>201423	203070±8016
UF1-E vs mutUF1-E				P=0.0007
EV vs LTAg				P=0.0001 (2.9x)
mUF1-E+EV	24350<br>24422<br>23670	0,211<br>0,219<br>0,189	115403<br>111516<br>125238	117386±7073
mUF1-E+LTAg	57555<br>44942<br>53875	0,215<br>0,202<br>0,180	267698<br>222485<br>299306	263163±38611
EV vs LTAg				P=0.0030 (2.2x)

Transfection 06.09.2017: UF1-L and mutUF1-L in HeLa

plasmid	LUC	protein	LUC/prot	Average±SD
UF1-L+EV	9146<br>10495<br>8338	0,154<br>0,205<br>0,158	59390<br>51195<br>52772	54452±4348
UF1-L+LTAg	482715<br>395586<br>330716	0,178<br>0,160<br>0,171	2711882<br>2472413<br>1934012	2372769±398393
UF1-L vs mutUF1-L				P=0.0641 (ns)
LTAg				P=0.0005 (44x)
mutUF1-L+EV	17254<br>11293<br>10346	0,138<br>0,145<br>0,130	125029<br>77883<br>79585	94166±26742
mutUF1-L+LTAg	90909<br>107943<br>63633	0,158<br>0,160<br>0,135	575373<br>674644<br>471356	573791±101653
LTAg				P=0.0014 (6.1x)

Transfection HeLa 08.09.2017

Early

plasmid	LUC	protein	LUC/prot	Average+SD
UF1-E+EV	20366<br>21480<br>18982	0,117<br>0,134<br>0,134	174068<br>160299<br>141657	158675+16266
UF1-E+LTAg	23635<br>20598<br>23230	0,126<br>0,097<br>0,105	187579<br>212351<br>221238	207056+17443
UF1-E vs mutUF1-E				P=0.7806
EV vs LTAg				P=0.0246 (1.3x)
mutUF1-E+EV	15203<br>15442<br>13077	0,088<br>0,100<br>0,096	172761<br>154420<br>136219	154467+18271
mutUF1-E+LTAg	23791<br>27015<br>21745	0,082<br>0,094<br>0,091	290134<br>287394<br>238956	271161+28038
UF1-E vs mutUF1-E				P=0.7806
EV vs LTAg				P=0.0038 (1.8x)

Late

plasmid	LUC	protein	LUC/prot	Average+SD
UF1-L+EV	7894<br>6523<br>7509	0,117<br>0,115<br>0,117	67470<br>56722<br>64179	62790+5507
UF1-L+LTAg	155364<br>284047<br>223066	0,118<br>0,140<br>0,141	1316644<br>2028907<br>1582028	1642526+359965
UF1-L vs mutUF1-L				P=0.0020
EV vs LTAg				P=0.0016 (26x)
mutUF1-L+EV	19324<br>14815<br>14988	0,117<br>0,116<br>0,106	165162<br>127716<br>141396	144758+18948
mutUF1-L+LTAg	31129<br>55505<br>30792	0,089<br>0,107<br>0,095	349764<br>518738<br>324126	412543+98517
EV vs LTAg				P=0.0099 (2.8x)

Transfection HeLa 13.09.2017

Early

plasmid	LUC	protein	LUC/prot	Average+SD
UF1-E+EV	22693<br>16286<br>21990	0,107<br>0,102<br>0,133	212084<br>159667<br>165338	179030+28766
UF1-E+LTAg	15729<br>21012<br>24778	0,088<br>0,110<br>0,149	178739<br>191018<br>166295	178684+12362
UF1-E vs mutUF1-E				P=0.0513
EV vs LTAg				P=0.9857 (0.998x)
mutUF1-E+EV	13195<br>10991<br>11531	0,093<br>0,101<br>0,090	141882<br>108822<br>128122	126275+16607
mutUF1-E+LTAg	34252<br>19590<br>24957	0,129<br>0,099<br>0,097	265519<br>197879<br>257289	240229+36906
EV vs LTAg				P=0.0082 (1.9x)

Late

plasmid	LUC	protein	LUC/prot	Average+SD
UF1-L+EV	7732<br>6295<br>6503	0,109<br>0,111<br>0,090	70936<br>56712<br>72256	66635+8619
UF1-L+LTAg	209056<br>202895<br>212890	0,129<br>0,099<br>0,097	1620589<br>2049444<br>2194742	1954925+298519
UF1-L vs mutUF1-L				P=0.0650
EV vs LTAg				P=0.0004 (29x)
mutUF1-L+EV	15531<br>9281<br>12672	0,100<br>0,105<br>0,115	155310<br>88390<br>110191	117964+34131
mutUF1-L+LTAg	56053<br>81420<br>51723	0,098<br>0,116<br>0,089	571969<br>701897<br>581157	618341+72507
EV vs LTAg				P=0.0004 (5.2x)

Transfection 15092017; HeLa cells; early promoter

plasmid	LUC	protein	LUC/prot	Average±SD
UF1-E+EV	9750<br>8590<br>8300	0,089<br>0,139<br>0,113	109551<br>61799<br>73451	81600±24897
UF1-E+LTAg	6754<br>6754<br>6203	0,087<br>0,082<br>0,072	77632<br>82366<br>86153	82050±4269
mutUF1-E+EV	5192<br>5271<br>5195	0,064<br>0,066<br>0,054	81125<br>79864<br>96204	85731±9092
mutUF1-E+LTAg	2481<br>2542<br>3016	0,055<br>0,058<br>0,072	45109<br>43828<br>41889	43609±1621
UF1-E vs mutUF1-E				P=0.8006
LTAg UF1-E				P=0.9769 (1.0x)
LTAg mutUF1-E				P=0.0014 (0.5x)

Late promoter

plasmid	LUC	protein	LUC/prot	Average±SD
UF1-L+EV	1945<br>1888<br>1704	0,065<br>0,060<br>0,057	29923<br>31467<br>29895	30428±900
UF1-L+LTAg	46663<br>34508<br>55637	0,081<br>0,075<br>0,071	576086<br>460107<br>783620	606604±163901
mutUF1-L+EV	5322<br>3734<br>3624	0,073<br>0,063<br>0,076	72904<br>59270<br>47684	59953±12624
mutUF1-L+LTAg	9875<br>9356<br>13076	0,063<br>0,058<br>0,071	156746<br>161310<br>184169	167408±14693
UF1-L vs mutUF1-L				P=0.0156
LTAg UF1-L				P=0.0037 (20x)
LTAg mutUF1-L				P=0.0007 (2.8x)
